# “It's Just Kind of This Thing That I Need to Navigate”: Young Women's Stories of Recoveries After Domestic Abuse in Childhood

**DOI:** 10.1177/10778012221125498

**Published:** 2022-09-15

**Authors:** Tanya Frances

**Affiliations:** 1 University of Stirling, Stirling, Scotland

**Keywords:** domestic abuse, recovery, narrative, young adulthood, childhood

## Abstract

Those who experience parental domestic abuse in childhood are affected in multiple ways, but existing research uses a narrow lens, relying on psychotherapeutic and neuroscientific understandings. This article uses a dialogical theory to explore women's recovery stories. Interviews were conducted with 10 women in England and a voice-centered narrative analysis was used. This article attends to gendered, psychotherapeutic, and neoliberal narrative resources that shaped participants’ stories. It concludes that recoveries after domestic abuse in childhood can be considered as dynamic processes that are individual, as well as shaped by social, political, and relational contexts that shape storytelling practices.

## Background

Domestic abuse has historically been conceptualized as an adult issue (Mullender et al., 2002), but a growing body of research continues to show that those who live with domestic abuse in childhood are directly impacted by these experiences in multiple ways (Callaghan et al., 2015; Katz, 2015; Överlien & Hydén, 2009). In this article, I explore the experiences of women who experienced parental domestic abuse in childhood. I use the term “experience” to describe what might otherwise be known as “witnessing,” “living with,” or “growing up with” parental domestic abuse. Parental domestic abuse refers to the intimate partner violence and abuse used between the participants’ parents, step-parents, or partners of parents. I use the term “experience” to recognize the direct way that children participate in their family lives and are therefore directly affected by their experiences of domestic abuse (Överlien & Hydén, 2009).

The prevalence of domestic abuse and the number of children affected by it is likely significantly underestimated by official sources of prevalence rates (Walby & Towers, 2017). This is partly due to low reporting rates (MacQueen & Norris, 2016) and insufficient methods of gathering data such as counting individual incidents, rather than capturing patterns of abuse and dynamics of controlling behaviours which characterize how domestic abuse is maintained (Walby & Towers, 2017). It remains unknown, exactly how many children and young people in the UK are affected by domestic abuse, but estimates suggest that 25% of young adults experienced domestic abuse in childhood (Radford et al., 2013). Concerningly, as funding decreases for sexual and domestic violence services in the UK (Hawkins & Taylor, 2015; Sanders-McDonagh et al., 2016), there are more women and families finding no refuge spaces available (Austin, 2021), and more child referrals to social care (Action for Children, 2019). Centralizing children and attending to childhood experiences of domestic abuse is an important concern, but despite calls for meaningful policy and strategy reform (Action for Children, 2019), children are still notably obscured in UK government strategy (HM Government, 2021).

Existing research highlights how children are adversely affected by domestic abuse, such as negative impacts on physical and mental health (Holt et al., 2008), emotional and social development (Fusco, 2017), and educational attainment (Byrne & Taylor, 2007). The question of what makes people resilient following exposure to domestic abuse threads through much of the literature, positioning people as either “resilient” or “not resilient” (see, e.g., Bowen, 2015; Howell, 2011). This categorization and either–or thinking does not account for complexity or context. It is shaped by a deficit model of development, (re)producing problematic assumptions about the “growing up” and recovery trajectories of those who experience domestic abuse in childhood.

There has been little attention to children's recovery trajectories following domestic abuse in childhood which does not provide a narrow and limiting picture of outcomes in adulthood. Several studies have examined interventions for children, including studies that centerthe experiences of children (Frances et al., 2019; Callaghan et al., 2019; Katz, 2015; Pernebo & Almqvist, 2016) and studies that focus on intervention effectiveness (Howarth et al., 2015; Lee et al., 2012; Smith et al., 2015). Additionally, several studies have explored factors that promote resilience (Anderson & Bang, 2012; Howell & Miller-Graff, 2014), though there is little centralization of the voices of those who have lived with domestic abuse, from a young adult retrospective perspective.

Notably, a small number of non-UK-based retrospective studies have been conducted with adults who experienced domestic abuse in childhood with a focus on resilience (Alaggia & Donohue, 2018; Anderson & Danis, 2006; Gonzales et al., 2012; Jenney et al., 2016; O’Brien et al., 2013). Whilst these studies are useful in centering the voices of those who have experienced domestic abuse, they are still justified by the assumption that some people are resilient, and others are not. A focus on resilience or coping limits the space for people to express how recoveries are experienced beyond factors contributing to resilience or coping. It sets up a binary way of thinking about resiliency (i.e., those who are resilient and those who are not, and there is no in-between, or very little possibility to occupy both positions). This binary framework is also based on limiting conceptualizations of resilience, often framed as an individual set of qualities or characteristics, with little attention paid to contextual or relational factors that might shape how people cope with and live after domestic abuse, and how this might change over time. This narrow focus on resilience or coping also limits what we can know about people's experiences, because if we only ask questions about resilience, it is likely that those are the stories people will share with us as researchers.

One recent study has explored the perspectives of young adults in Canada with a broader focus on meanings assigned to childhood experiences of domestic abuse, rather than specifically on resilience or coping, offering a significant contribution to the literature (Dumont & Lessard, 2019). Their work provides an important divergence from the existing literature as it does not center around coping or resilience. Using a life course approach, they explored the experiences of young adults, and they suggest that developmental transitions in recovery from domestic abuse, consist of multiple individual and relational factors for young adults over the life course. They suggest that the meanings young adults gave to their experiences of domestic abuse were shaped by experiences of school, work, friendships, and other significant relationships, and these meanings relied on awareness, developing trust in other people, and a sense of becoming empowered. These findings are important, and demonstrate that relational, environmental, and contextual factors can shape how young adults make meaning out of their childhood experiences of domestic abuse. It also demonstrates that the theories researchers use, and the questions we ask, play a significant role in shaping what participants say and how we make sense of the accounts that are shared (Sermijn et al., 2008).

Understanding domestic abuse recoveries through an outcomes-focused individualized lens presents troubling narrative frameworks that privilege a single linear story of resilience, or inevitable “damage” caused by living with domestic abuse. The homogenization and individualization of outcomes work against a multidimensional and relational way of understanding how recoveries from domestic abuse in childhood are experienced, specifically in relation to transitions to young adulthood. In this article, I use a dialogical theory (Hermans, 2001) to examine the way that neoliberal narrative resources play a part in shaping how women narrate recovery stories. In doing this, I refer to neoliberal ideologies as the individualization and self-responsibilization of self-improvement, “success,” and happiness (Alcoff & Gray, 1993; Rose, 1985). This study addresses these issues by exploring young adult women's accounts of recoveries after domestic abuse in childhood. I have pluralized the term “recoveries” to reflect participants’ multiple stories and the diversity of their experiences of domestic abuse. The pluralization of these terms also functions to reject the homogenization of experiences and the erasure of stories that do not align with dominant narrative resources about recovery or growing up.

## Methods

A dialogical narrative approach (Frank, 2010; Hermans, 2001) was used to explore women's accounts of their transitions to young adulthood after experiencing domestic abuse in childhood. Narrative inquiry is theoretically diverse, fluid, and flexible (Livholts & Tamboukou, 2015), and it was particularly useful for this study in its capacity to centralize “voice(s)” and highlight relationality, context, power, and lived, storied experiences (Riessman, 2008). The starting point was that lives are storied, and it is through stories that we make meaning out of our experiences and construct a sense of self (Frank, 2010).

Ten women aged 21–35 in England participated in narrative interviews that were conducted by myself. The women who participated had all experienced domestic abuse in childhood, and most participants had experienced other forms of abuse too, such as direct physical, sexual, and emotional abuse in addition to parental domestic abuse. All participants had not accessed any formal source of meaningful support to address their experiences of domestic abuse as a child. I provided participants with an interview topic guide in advance of the interview, and I used an open narrative interviewing style guided by the topics on the topic guide. Women were invited to share experiences of their childhoods, their transitions to young adulthood, and their sense of what their experiences of domestic abuse meant to them. Interviews lasted between 75 and 135 min, and took place in various locations, including on Skype, on the phone, in university interview rooms, university library rooms, or participants’ homes, depending on participant preference.

Interviews were transcribed and the listening guide (Brown & Gilligan, 1993; Gilligan, 2015) was used as part of the analytical process. The listening guide is a feminist narrative voice-centered method (Doucet & Mauthner, 2008; Gilligan, 2015) consisting of three “listenings,” resulting in the production of “I Poems,” representing shifts in multiple 'contrapuntal' voices that threaded through participants’ accounts. A more detailed account of how I worked with the material and engaged with the poems can be found in a methodological paper published earlier (Frances, 2022). The listening guide spoke well to a dialogical theory of selfhood (Hermans, 2001), recognizing that the self is not a single unitary subject, but the self is constructed of multiple 'voices' and selves, which exist in dialogue with each other, and are each situated and shaped by the person's individual biography as well as the social, cultural, and relational contexts which they live in and in which they give voice to their stories. This dialogical approach to narrative analysis attended to selfhood and voice as multiple, contextual and relational. This attention to polyvocality and multiplicity was an important methodological and ontological commitment for this study, because often when women talk about abuse or traumatic experiences, their accounts risk being flattened out to a single storyline, neglecting the multiplicity of their stories and identities (Alcoff, 2018; Woodiwiss, 2007). From a feminist standpoint, this meant assuming that storied lives are multiple and consist of entanglements of the personal and political (Thompson et al., 2018). It was therefore important to attend to individual stories, as well as the social, political, and relational contexts in which the stories were told in. This study received ethical approval from the University of Stirling General University Ethics Committee.

## Recoveries After Domestic Abuse

Participants narrated their recoveries through stories of strength, struggle, resilience, and “moving on.” Their accounts were shaped by psychotherapeutic self-evaluative structures and neoliberal gendered ideologies. This article explores how these recovery stories can be useful to young women, but I also explore how existing narrative frameworks may simultaneously constrain which recovery stories are speakable, how they are told, and may also limit how they are heard. Case examples are provided throughout the following sections. Not all participant interviews are drawn on in tihs article, as I wanted to provide depth rather than breadth of analysis, and as such, it felt important to maintain the context of the participant and the interview. Whilst I do not draw on all participants in these sections, the analysis of the recovery stories I provide examples of does reflect how I made sense of most of the participants’ accounts and my analysis of the accounts reflect common themes and ideas that threaded through my analysis of the stories I worked with. This article focuses on storytelling practices where recovery stories were shaped by psychotherapeutic narrative resources and gendered neoliberal ideologies.. Names used in this article are pseudonyms, and any potentially identifiable information has been changed.

### Storying Recovery Through a Psychotherapeutic Gaze

In narrating their recoveries, participants’ accounts highlight realizations that they were struggling, realizing their childhood was not “normal” or piecing together parts of their lives in ways that they had not done before. What I have termed as “the psychotherapeutic gaze,” refers to the way that psychotherapy, psychiatry, and a neuroscientific lens, have power in offering an authorized account through which young women could talk about their experiences of domestic abuse and their recoveries. Here I turn attention to narrative practices where participants turned their gaze inwards. For example, participants asked what it was about themselves that they could change, diagnose or understand, for things to be different or better. A self-reflective inward-facing gaze was one way in which young women narrated their recovery stories and made meaning out of what had happened in their lives. It was also an important way of enabling women to story a self that has moved on and recovered. For example, Hayley highlighted the likelihood that she would be more “at risk” of mental illness, stating that: “I had more risk factors that I was going to choose to go down the path that I went, therefore there was more chaos during my adolescence and there was more risk of mental illness.”

Hayley reflected on why she struggled with her mental health, explaining that she grew up on a poor council estate, had young parents, and was around drugs and alcohol from a young age, in addition to parental domestic abuse. Through her account, she turned the gaze inwards by using statements such as “I had more risk factors… I was going to choose to go down the path that I went.” This is a self-evaluative structure using expert language such as “risk factors.” This self-evaluative structure points to psychotherapeutic and psychiatric discourses that promote a self-reflective and internalized gaze (Rose, 2010).

Liv's narration also points toward self-evaluative structures as she spoke about the “black mark” that she felt she carried.

I feel like my whole childhood is like a black mark and people can see it. Itmakes me feel dirty and like, different… I really struggle with relationships and friendships and stuff. I’m actually going through an assessment for Asperger's because I feel like, I don't know [pause] I feel like I do score highly on all the tests and stuff and I don't know if it's that, or if it's all the trauma that makes me this way. (Liv)

Liv's uncertainty, “I don’t know if it’s that, or if it’s all the trauma” points to the challenge she faced in narrating a self that is coherent and stable. Efforts to understand herself by making links between childhood trauma and adulthood difficulties threaded through Liv's storytelling practices. Liv made sense of her current struggles by linking them to her past trauma. This inward gaze shows her efforts at making sense of her childhood, but her narrations show that making sense of her childhood is not straightforward. A diagnostic and psychotherapeutic framework enabled possible alternative constructions of the self that were not led by self-blame and shame and that enabled a legitimized way of making sense of her struggles, such as the 'assessment for Asperger's' or her proposal that maybe it is 'all the trauma'. Telling her recovery story through a self-evaluative structure (Alcoff & Gray, 1993) made possible a story in which at least her struggles can be recognized and her own sense of shame might be lightened. However, the “black mark” constructs the self as “damaged” underpinned by the sense that this damage is a visible and dark mark of difference. Thisvoice of shame and damage was shaped by a powerful narrative resource of the trajectory of inevitable damage that people who grow up with domestic abuse are assumed to experience.

Liv did not know if it is “that” (i.e., Asperger's), or “all the trauma” that caused her struggles in young adulthood. These individualizing psychotherapeutic narrative frameworks also shaped the way that Emma made sense of her difficulties.

Emma: I kind of referred myself for an ADHD diagnosis and cos as time's gone on I’ve kind of learnt about myself and about ADHD and I kind of (pause) don't (pause) really see it in that way (pause).

Int: in what way?

Emma: I don't want to say (pause)—like I can kind of rationalise my struggles (pause) as something you know – (pause) I just find it interesting that I had to pathologise myself (slight laughter) to give myself an OK, and a reason why I do these things, a reason that I get anxious and a reason that I behave in this way. It's because I’ve got ADHD. But actually I think now I realise (pause) well I’m trying to remind myself (pause) that maybe I’ve just had some hard experiences that have made me – that have moulded me in that way.

In Emma's account, there is a voice of self-acceptance, for example, “I’m trying to remind myself that maybe I’ve just had some hard experiences.” There is also a voice shaped by a psychotherapeutic and medicalized lens. For example, “it's because I’ve got ADHD.” At the same time, there is also a voice that rejects self-evaluation from a psychotherapeutic gaze. Emma said, “as time's gone on I’ve kind of learnt about myself and about ADHD and I kind of don't really see it in that way.” She also said, “I find it interesting that I had to pathologise myself….” From this view, whilst Emma rejected a psychotherapeutic discourse, a diagnosis also offered her a sense of empowerment. An “expert” story functioned to authorize Emma's struggles in young adulthood, making her story credible and readable to others, bolstering the acceptance she could give to herself. As Woodiwiss (2014) has also explored with women who had experienced childhood sexual abuse, this pathologization had been Emma's route to understanding herself. Through the same account, there also exists a rejection of the fact that she had to pathologize herself, pointing to the tension in narrating the reasons for her struggles and the challenge of telling a story that renders her recovery credible.

Emma's negotiation of these tensions continued, shaped by an underpinning gaze toward the self. She explained, “I think I’m learning to be a bit kinder to myself and think, you know I didn't experience you know a rape you know, or an event, but I saw lots of little things over a long period of time.” She then explained, “I have to remind myself that that can be quite traumatising.” The voice of self-compassion (“I’m learning to be a bit kinder to myself”) is shaped by psychotherapeutic narrative resources of recovery and healing. Telling a story through a self-evaluative structure enabled Emma to link the past to the present in a way that made sense (Woodiwiss, 2014), stabilizing her story, and as such, stabilizing the self through expert discourses. Psychotherapeutic self-evaluative structures may also enable the possibility for the self to be constructed as someone deserving of kindness and empathy, by her recognition: “I’m learning to be kinder to myself” and “I’ve held onto it for so long and it tears you apart.” Emma's recovery story told through this psychotherapeutic inward gaze was powerful in enabling Emma to reconstruct herself as someone who was not responsible for her struggles; she owns them, but through this self-evaluative recovery story, she is not responsible for them.

### Narrating a Stable and Credible Recovery Story

Recovery stories included accounts of resilience and moving on. These stories enabled women to write themselves into a different kind of life than the one that they had experienced in childhood, but there were narrative challenges in voicing emotion, particularly emotion that risks destabilizing a coherent and stable recovery story. A central part of Frances’ recovery story was her pride that she had chosen the “better life” and shown resilience in doing so. In Frances’ childhood, she had disclosed the abuse she experienced to teachers and had social workers visit her home, but nothing was ever done, and nobody believed her when she disclosed it.

It just kind of shines a light to me about how resilient I am and how proud I am of myself. But then talking about it, and reflecting on it with you, has started like a bit of an anger fireball going, where I just think how did I get let down so badly? How did I get ignored by the people that could have got me out of that situation so many times? And it makes me feel sad for other people that may have experienced similar things to me but they didn't have the resilience to choose the better life. And they would have just been let down by all these people and by the system… Erm you know, potentially revisiting kind of like the social services that let me down is something that I have thought about, you know… But for me to do that I need to know that—I need to be ready to do that, and right now I’m not ready to do that. (Frances)

Through Frances’ account of moving on in her recovery, she told a story of pride and resilience. However, there also exists a voice of anger that does not always align with a stable and credible account of resilience.

I would never want anyone to experience what I went through or the feelings that I had to endure or the behaviour that I was subjected to. I’d never want anyone to go through that. And the way I could do it is by going back to the services and giving them some feedback. But then on the flipside I just think, well what‘s the point? They let me down before, they’re not gonna take my feedback seriously. And that trust isn't there. And that almost kind of—on their part I don't want to cooperate with them because I’m angry with them, but then I want to be able to help others. So it‘s just kind of this thing that I need to navigate. (Frances)

Frances suggested she wants to make sure others do not experience the same as her. Using her experience for good in order to help others is framed as central to a recovery story in which she has chosen “the better life.” However, a story of resilience and recovery is at odds with the anger that is also voiced. Anger is a voice that becomes less speakable, existing on the margins, or becoming silenced in favor of an account of resilience. This resilience enables space for a certain version of recovery but one in which she is “not ready” to revisit services because she is angry. Through her account, Frances positioned herself as an adult with knowledge about “how the world works.” Through this positioning, she expressed her anger with anticipation that it would be dismissed. On the one hand, she knew that her anger meant she is less likely to be heard. However, she also felt a responsibility to her memory, to herself, and to others, to provide the service with some feedback in the hopes that what happened to her would not be repeated, producing a tension in her storytelling.

Frances did not feel she could put her anger to use because she was “too” angry—she was not ready and did not see the point. Anger is storied as raw emotion, unprocessed, and as producing a sense of incoherency. Emotion—anger, in this case, is not just individual but is politically and socially constituted, produced, and expressed (Ahmed, 2014; Alcoff & Gray, 1993). Frances’ sense that she was “too angry” to speak points to the power at play in the sociocultural context that shaped how she told her story. Using her experience to do good, by speaking out and helping others is part of a neoliberal recovery story that allowed her to talk about gaining strength and empowerment. However, Frances’ recovery story is regulated and shaped by by neoliberal ideologies that risk silencing the anger she carries—so much so, that at some points, her anger drowned her capacity to articulate it in a linear and neat way which might have been readable by me in the interview context, leading her to conclude, “it's just kind of this thing I need to navigate.”

Anger operates within power relations that are gendered. If women speak with “too much” emotion, it is said to “transgress appropriate survivor talk” leading women to police themselves and be policed in relation to their emotions (Alcoff & Gray, 1993, p. 285). Frances’ anger at the way she had been let down by services motivated her to consider going back to services to give them feedback in the hopes it would help others. Frances also voiced ambivalence about how her anger is expressed and used. There can be particular ambivalences for women about how anger is expressed and used, because anger is not always considered synonymous with femininity (Holmes, 2004). Others have suggested that women expressing anger on their own behalf can function to pose a threat to a patriarchal society where women are generally invited to stay small and anger is a typically “masculine” expression (Alcoff & Gray, 1993). Thus, through a neoliberal recovery story, women might be more able to express anger on behalf of others, as expressing anger on behalf of others rather than on behalf of the self, is a more “appropriate” and acceptable response to violence and it is a recognized and credible story of moving on. Considered in this way, Frances’ proposal to go back to services so that the same does not happen to others is central to her recovery story and functions to stabilize a story that may be destabilized by expressing anger on behalf of herself.

These narrative resources of credibility, femininity, and emotion also intersect with Frances’ sense of pride that she chose the “better life” and had the resilience to do so. Some participants spoke about being OK enough to tell their stories—about having done the work to enable them to feel OK with talking openly about their experiences. Others said the interview was the first time that they had spoken openly about their experiences, but they felt a responsibility to contribute to research to help others. Regardless of how participants framed their capacity and motivation to share their stories, a sense of having moved through to the other side, a sense of having “traveled through,” puts their struggles in the past and supports the narrative construction of the self as being self-knowledgeable and having survived. A survival position is not explicitly named as “survivor” in participants’ accounts, but the way that participants narrated recovery, resilience, self-development, and “traveling through” does align with dominant discourses that surround survivorship (Alcoff & Gray, 1993; Orgad, 2009; Ovenden, 2012). Survival stories can be empowering. They can provide a framework for talking about trauma and recovery in a way that can be heard by others and it can help to construct the self as resilient. However, stories that construct the self as a survivor can simultaneously be limiting (Alcoff & Gray, 1993; Reich, 2002). Thus, struggles can be difficult to articulate because of the lack of favorable and supportive narrative frameworks through which to tell them.

### Forgiving, Forgetting, and Moving on: Neoliberal Recovery Stories

Recovery stories were also shaped by the self-development work required to use inner strength and “move on.” For example, Jasmine spoke about gaining strength from her experiences, explaining that “in order to forget I’ve kind of gotta forgive what happened… forgive what I was put through and accept that it was part of life.” Jasmine reflected that “that did happen to me but it doesn‘t necessarily mean that my whole life has to be ruined.” Through her account she explained the self-development work she did in order to forgive, forget, and move on. These stories of healing, strength, and forgiveness functioned to establish her as no longer impacted by the violence she grew up with. I asked Jasmine what it was like to reflect on her childhood.

Because I have dealt with it, it always feels like I’m talking about someone else, which is really weird. [Int: OK] it's just something that I think, because I’m so at peace with it now, it's just something that kind of happened, like I brushed my teeth yesterday, like I brushed my teeth this morning. It's just something that happened that is just [pause] part of who I am. (Jasmine)

Jasmine established a sense of narrative distance, positioning childhood as something she is no longer impacted by; it is just “something that happened.”

When I’m talking about it I picture myself as a little girl, and that's who I’m talking about. And obviously I know that I am that little girl, but it's kind of like all of that happened to a little girl and that isn't me. Although it is—but it's just like I’ve lived like two lives. (Jasmine)

This gap between experiencing (“it's kind of like all that happened to a little girl and that isn't me”) and knowledge (“I know I am that little girl”) is something that Hydén (2014) notes is common for people who have previously been victimized. She suggests that telling stories that help to establish a sense of distance can be a form of psychological protection against overwhelming pain. A sense of distance can be understood as having a narrative function that does something useful for Jasmine, by establishing a sense of psychological safety. However, distance and disconnect do not align well with the autobiographical coherence and connectedness of the therapeutic recovered self that requires a story that can connect the past to the present in a linear and coherent way. At the same time, this distance helps to position Jasmine as secure in her adult identity and affords her epistemic privilege—a position where she is more likely to be considered a trustworthy source of knowledge (Alcoff, 1991; Fricker, 2007), bolstering the strength and wisdom that her recovery story writes her into. She reflected: “I think I’m definitely stronger, wiser—erm, but I still have inner conflicts about that because I sometimes wanna be that little girl again.”

Jasmine's acknowledgment of these conflicts suggests there is a challenge in narrating the story of herself as a child that her adult self does not identify with, yet which she knows is part of her life. A gap between knowing and experiencing serves a useful function by offering safety and a sense of having navigated a transition to adulthood “successfully.” However, that knowledge–experience gap is also difficult to narrate as it leaves gaps. It prompts questions of where and how stories of childhood, violence, struggle, and instability are able to be told and heard. These are experiences that victimized her, and as such, they are not culturally valuable stories to tell. As Hydén (2014) reflects, they are stories that tend to position people as unlovable and invaluable, and these can be unspeakable stories to tell. Being an adult is therefore not a straightforward, single, or unitary position to write herself into. Jasmine's articulation, “I know I am that little girl,” but “that little girl isn't me,” followed by the knowledge that “it is” her, illustrates this tussle and suggests that recovery, rather than being linear, could be considered fluid, dynamic and even interrupted or fragmented in places. Jasmine reflected that she had “dealt with her demons,” but her conflicting feelings were not entirely left in her childhood. Jasmine explained that some of these questions still exist now, thus, that sense of narrative distance is not always possible.

I used to think and I still kind of think he put me through that and stuff because I looked like my mum. So I used to think that he struggled with me because he’d look at me and see my mum. I used to think that that was obviously my fault—I used to think that it was me. (Jasmine)

Jasmine has put her “demons” behind her, but she still “kind of thinks” it was her fault. This hesitancy serves to break that sense of narrative distance and suggests that recovery is not as simple as “moving on,” but it is fluid, dynamic, and consists of conflicts that may get erased through the telling of linear stories that do not account for fluidity and interchanging positions. Jasmine's story of becoming stronger, older, and wiser is supported by narrative resources of adulthood and resilience that have a particular kind of social power for women who have experienced trauma (Reich, 2002). When women speak and tell stories of surviving, these are socially valued stories; they position women as strong, resourceful, and successful (Alcoff & Gray, 1993; Reich, 2002). As such, these stories are useful in enabling a sense of empowerment. However, these stories do not always enable the articulation of tensions that are not only located and left in childhood, but which exist in adulthood too.

## Making Sense of Coexisting and Multiple Recovery Stories

This article so far has highlighted the multiple and coexisting recovery stories women told, paying particular attention to the way that individual stories intersect with, and are told through, gendered and neoliberal social structures. This section unpacks the themes that run through these accounts of recovery, offering an understanding of women's stories and storytelling practices around recovery after childhood domestic abuse that is attentive to multivocality, relationality, and context.

### Telling an Authorized and Legitimized Recovery Story

Participants had not accessed services or received support to address their experiences of domestic abuse. Given that their childhood experiences of domestic abuse took place out of the gaze of services or institutions which might have validated and legitimized their experiences, there are limited ways of talking about it or even naming it as domestic abuse. Previous research has explored the power of an authorized account of domestic abuse, suggesting that childhood accounts can be shaped by professionalized or therapeutic discourses, as these accounts are readable and accepted versions of the violence that happened (Callaghan et al., 2017). These authorized accounts do not always fit with experiences of abuse, and they serve to smoothen out the multivocality of the expression of their experiences. However, authorized accounts also have the capacity to provide a stable story that is more likely to be considered reliable by those who are listening (Callaghan et al., 2017).

A language and discourse of experiences of trauma and the effects of trauma, has been popularized in social and cultural discourse, through survivor movements such as #MeToo and the increase of trauma-informed and trauma-sensitive awareness across communities and health and social care settings (Alcoff, 2018; Elliott et al., 2005; Walkley & Cox, 2013; Zaleski et al., 2016). The popularization of the language and understanding of trauma may have strengthened the survivor discourse that shaped recovery stories. However, it also limits what can be said and it some settings, narrating the self as survivor may put pressure on people to disclose in a certain way or before they may feel ready. This speaks to the way that theory-based knowledge is privileged when people speak about the abuse they have experienced (Alcoff & Gray, 1993). For example, Frances storied herself as a person who has gained resilience and strength from her experiences, positioning her in a survivor position, despite the ways that her experiences transgress dominant survivor narrative frameworks due to her ambivalence about using her anger to speak out. This highlights that participants whose stories have not been verified or validated by those with theoretical expertise or social power are left in an epistemic gap where their experiential knowledge is not always counted in all spaces, and has limited ways of being told.

### Narrating and Resisting the Self as a Therapeutic Subject

Narratives of self-improvement were told in a neoliberal context that privileges self-driven success and happiness. These are culturally valuable stories to tell particularly when occupying a survivor identity (Alcoff & Gray, 1993; Rose, 1985). Neoliberal ideologies promote a self-evaluative structure in which the self is constructed as a therapeutic subject, and the “work” of recovery is to work on the self “successfully” (Woodiwiss, 2014). This kind of neoliberal story enables a coherent narrative, and it offers ways of making sense of distress whilst also offering solutions (i.e., therapy, medication, or other forms of healing). For example, the tension of whether Emma's struggles are because she has attention-deficit hyperactivity disorder (ADHD) or because of trauma demonstrates a search for a reason for her struggles so that she can better understand herself and move on. The existence of these narrative resources of self-improvement and self-driven success can be useful and can help women to move through, survive and construct a sense of self that has the capacity to change and the power to do so.

This self-evaluative structure, shaped by an individualizing and psychotherapeutic way of making sense of distress can be useful, but it also functions to decontextualize people's struggles and distress. For example, Emma's quest to understand whether her distress was because of ADHD or “all the trauma” points to this self-evaluation and individualization. These explanations also offer a “neat” and coherent narrative that has logic and therefore renders her story readable to those she shares it with. A psychotherapeutic lens that centers on a need for a coherent way to make sense of and locate the source of distress also promotes a neuroscientific way of conceptualizing resilience due to the individualizing and often diagnosis-led quest to find a meaning and make sense of distress. This neuroscientific conceptualization of resilience refers to the way that resilience can be understood as an individual trait or quality that is located within the brain (Macvarish et al., 2015; Rose, 2010; Wastell & White, 2012). Individualizing assumptions such as this underpin much of the psy-professions’ (i.e., psychology, psychotherapy, and psychiatry) way of understanding how trauma can have immediate and long-lasting impacts on people (e.g., Rothschild, 2017), and these neuroscientific conceptualizations can be helpful, in shaping what we know about how people respond to trauma and how people can be supported to recover. However, neuroscience alone is not enough. Critiques of the neurodevelopmental discourse are not new (Burman, 2017; Featherstone et al., 2014; Rose, 2010; Wastell & White, 2012). It has even been critiqued from within neuroscience itself, as neuropsychologists have questioned the extent to which we have theorized regions of the brain, showing we do not know as much as we think we know (Uttal, 2011).

This individualizing “resilient brain” discourse (Rose, 2010) also promotes a concerning conceptualization of risk culture, whereby “risk” becomes no longer a person, situation or context which *poses* a risk (i.e., in this context, the person who perpetrated violence or the sociopolitical structures which enabled the violence), but the *person themselves* becomes the risk, and consequently, the individual becomes something to fix (Burman, 2017; Rose, 2010). The sole use of neuroscience as evidence remains unchallenged in mainstream practice meaning that neuroscience discourses about resilient brains continue to be pervasive and continue to reproduce assumptions that resilience and wellness in adulthood is an individual trait. I have explored how this can be seen in participants’ accounts, where in various ways, participants demonstrated a self-reliance, a self-evaluation, and a focus on the self to “move on,” “forgive and forget” or become “stronger” and “wiser” or “choose the better life.” While I have explored how these stories are useful to participants in offering a sense of empowerment, these accounts are also shaped by neoliberal individualizing understandings of “successful” recovery, and they offer little space for attending to the social, political, and relational contexts that people recover in, and which shape how people make sense of who they are and their experiences of violence or abuse (Gill & Scharff, 2011; Rose, 2010; Wastell & White, 2012).

Neoliberal values of self-improvement and self-responsibility pave the way for what kind of recoveries are possible to talk about and how, and this intersects with gender in important ways. Feminist scholars have argued that contemporary northern narrative frameworks locate femininities or womanhood within individualizing discourses of self-work and self-improvement (Burman, 2017; Gill & Scharff, 2011; McRobbie, 2004). In relation to women's recoveries from domestic abuse, a psychotherapeutic discourse not only suggests that “we all have the right to personal happiness, success and satisfaction, but direct ourselves to construct ourselves as damaged and ultimately responsible if we do not live such lives” (Woodiwiss et al., 2017, p. 16). As such, neoliberal values surround a particular kind of femininity whereby a successful and speakable recovery is self-made and self-driven.

Neoliberal and gendered social structures operate in ways that can be both limiting and empowering. Telling a neoliberal recovery story that does not contain “too much” emotion or struggle is useful; it can provide a quality of coherency that has the capacity to stabilize the therapeutic recovered self. However, this risks women self-narrating stories in which they alone are responsible for their happiness and recovery, erasing the social, relational, and political ways in which recovery and violence are located (Rose, 2010; Wastell & White, 2012). Individualizing psychotherapeutic narrative frameworks can invite all adulthood difficulties to be correlated with the abuse experienced in childhood, and this can limit the way that recovery stories can be articulated. These individualizing neoliberal narrative frameworks invited participants to conclude that it must be something about *them* that they should work on in order to “do” recovery well, as opposed to storying recovery in a way that recognizes the role that relational, social, and political factors might play in shaping what kinds of recoveries are possible and what kinds of recoveries become possible to speak about.

### Attending to Multivocality

Using a dialogical theory (Hermans, 2001) to attend to the polyvocality of participants’ stories was an important methodological and ontological practice. This practice enabled me to reject a single unitary self logic, assuming that “I” is stable, authentic, and singular, and work with the idea of the self as multiple, fluid, and expressed polyvocally (Sermijn et al., 2008). Through this way of working with stories, I have explored the idea that recovery stories were not fixed, and they consisted of nuances and marginalized voices such as shame, doubt, uncertainty, loss, and hope. However, articulating these nuances risks producing incoherence in a narrative, destabilizing the recovery story, and producing an unstable “I.”

From this view, the assumption that recovery is linear and that the endpoint is fixed does not align with how participants narrated their stories. Stories of getting by, moving on, and surviving were messy and sometimes hard to articulate and challenging to make sense of, as a listener trying to engage with and make sense of participants' accounts. For example, Jasmine's position as having moved on was central to her articulating her strength and resilience, but that positioning left little space to express the uncertainties and conflicts she still held. Drawing on this example, recovery stories were relational and situated, meaning that when we tell our stories, and when we listen to the stories of others, these are not neutral spaces (Lenz Taguchi, 2012). In Jasmine's example, her story was told in a sociopolitical context that values a particular kind of recovery story that is linear, stable, and has a fixed endpoint of strength and survivorship (Alcoff, 2018). As such, Jasmine's account was told through a story of “moving on” and being “stronger and wiser,” though as explored, this story of “successful” recovery made little room for conflict, struggle, or tension without unsettling the linearity and stability of the story. In line with a dialogical philosophy, the self is always unfinalized, and how we story the self is always situated within the context of the telling (Hermans, 2001), therefore the “I” is not single, but it is multiple. Jasmine's story was one of strength and survivorship but through attending to multivocality I also explored how it was entangled with stories of struggle. In the context of recovery stories such as the ones I explore in this article, this means that the recovered self only comes into existence through dialogue and relationships. Therefore, how we come to understand recoveries and theorize the process of recovery, is as much about attending to the way the *context* of the telling shapes the stories that are told, as well as attending to the individual speaker themselves.

## Conclusions and Implications

Women's stories were shaped by psychotherapeutic, neoliberal, and gendered narrative resources that were both useful and limiting. These narrative resources offered a sense of resilience, strength, and a way of storying the self into a socially acceptable, legitimized, and even desirable self-made successful recovery story. Participants used a self-evaluative structure, constructing the self as a neoliberal therapeutic subject, and constructing recovery from domestic abuse as an individual, self-determining piece of work. However, there are narrative challenges that women faced in narrating a coherent and authorized therapeutic recovered self. Psychotherapeutic discourses about recovery from domestic abuse are shaped by assumptions that people recover in a linear way, suggesting that there is an endpoint to a recovery that is fixed and measurable. This recovery discourse is powerful and invites people to write themselves into a story where there is an endpoint to the recovery process. Narrating recovery as linear can be useful because it provides a credible and authorized way to tell a story of recovery, but it is also limiting as it leaves little space to also tell coexisting stories of struggle, tension and contradiction.

How people tell their recovery stories is shaped by the time and context of the telling, including how we anticipate the listener to receive and hear what we say (Brison, 2002). As such, drawing on a dialogical theory, women's recoveries after domestic abuse in childhood can be considered as not a product or endpoint, but as a dialogical, situational, and relational process whereby the “I” is fluid, unfinalizable, and multiple. Existing narrative frameworks do not provide diverse enough, or appropriate frameworks within which young women can make sense of their experiences of childhood domestic abuse. Dominant recovery narrative frameworks do something useful for women in providing a coherent story that has a certain kind of logic and that carries social value, but these frameworks also put significant limitations on what kind of story of recovery is possible to tell and how it will be heard. A broader definition of recovery is needed that accounts for fluidity, context, and change during the lifespan.

We also need domestic abuse policies, legislation, and practices that recognize people's relational and social contexts. This can be done by extending beyond “resilient brain” discourses that pervade the psy-professions and govern knowledge production and professional practice (Rose, 2010). These policies and practices should account for the sociostructural conditions that enable violence, and importantly, the social, structural, and relational conditions that can help people to recover and make meaning out of their experiences in a way that is useful to them (Frances et al., 2021). This article invites researchers and practitioners to consider how they work meaningfully with ambiguities and tensions, and how they listen to and work with human experience that is multiple and polyvocal and stories that are not always linear.

## References

[bibr1-10778012221125498] Action for Children. (2019). Patchy, piecemeal and precarious: Support for children affected by domestic abuse*.* Action for Children.

[bibr2-10778012221125498] Ahmed S. (2014). The cultural politics of emotion (2nd ed.). Edinburgh University Press.

[bibr3-10778012221125498] Alaggia R. Donohue M. (2018). Take these broken wings and learn to fly: Applying resilience concepts to practice with children and youth exposed to intimate partner violence. Smith College Studies in Social Work, 88(1), 20–38. 10.1080/00377317.2018.1404282

[bibr4-10778012221125498] Alcoff L. (1991). The problem of speaking for others. Cultural Critique, 20, 5–32. 10.2307/1354221

[bibr5-10778012221125498] Alcoff L. (2018). Rape and resistance. Polity Press.

[bibr6-10778012221125498] Alcoff L. Gray L. (1993). Survivor discourse: Transgression or recuperation? Signs: Journal of Women in Culture and Society, 18(2), 260–290. 10.1086/494793

[bibr7-10778012221125498] Anderson K. M. Bang E.-J. (2012). Assessing PTSD and resilience for females who during childhood were exposed to domestic violence. Child & Family Social Work, 17(1), 55–65. 10.1111/j.1365-2206.2011.00772.x

[bibr8-10778012221125498] Anderson K. M. Danis F. S. (2006). Adult daughters of battered women: Resistance and resilience in the face of danger. Affilia – Journal of Women and Social Work, 21(4), 419–432. 10.1177/0886109906292130

[bibr9-10778012221125498] Austin J. (2021). Nowhere to Turn 2021. Women's Aid Federation of England. Retrieved from September 5, https://www.womensaid.org.uk/womens-aid-launches-nowhere-to-turn-2021-report-findings-from-the-fifth-year-of-the-no-woman-turned-away-project/

[bibr13-10778012221125498] Bowen E. (2015). The impact of intimate partner violence on preschool children’s peer problems: An analysis of risk and protective factors. Child Abuse & Neglect, 50, 141–150. 10.1016/j.chiabu.2015.09.005 26410625 PMC4685964

[bibr14-10778012221125498] Brison S. (2002). Aftermath: Violence and the remaking of a self. Princeton University Press.

[bibr15-10778012221125498] Brown L. M. Gilligan C. (1993). Meeting at the crossroads: Women’s psychology and girls’ development. Feminism & Psychology, 3(1), 11–35. 10.1177/0959353593031002

[bibr16-10778012221125498] Burman E. (2017). Deconstructing developmental psychology (3rd ed.). Routledge.

[bibr17-10778012221125498] Byrne M. D. Taylor B. (2007). Children at risk from domestic violence and their educational attainment: Perspectives of education welfare officers, social workers and teachers. Childcare in Practice, 13(3), 185–201. 10.1080/13575270701353465

[bibr18-10778012221125498] Callaghan J. E. M. Alexander J. H. Sixsmith J. Fellin L. C. (2015). Beyond “witnessing”: Children’s experiences of coercive control in domestic violence and abuse. Journal of Interpersonal Violence, 33(10), 1551–1581. 10.1177/0886260515618946 26663742

[bibr19-10778012221125498] Callaghan J. E. M. Fellin L. C. Alexander J. H. (2019). Promoting resilience and agency in children and young people who have experienced domestic violence and abuse: The “MPOWER” intervention. Journal of Family Violence, 34(6), 521–537. 10.1007/s10896-018-0025-x

[bibr20-10778012221125498] Callaghan J. E. M. Fellin L. C. Mavrou S. Alexander J. Sixsmith J. (2017). The management of disclosure in children’s accounts of domestic violence: Practices of telling and not telling. Journal of Child and Family Studies, 26(12), 3370–3387. 10.1007/s10826-017-0832-3 29176928 PMC5682866

[bibr21-10778012221125498] Doucet A. Mauthner N. S. (2008). What can be known and how? Narrated subjects and the listening guide. Qualitative Research, 8(3), 399–409. 10.1177/1468794106093636

[bibr22-10778012221125498] Dumont A. Lessard G. (2019). Young adults exposed to intimate partner violence in childhood: The qualitative meanings of this experience. Journal of Family Violence, 35, 781–792. 10.1007/s10896-019-00100-z

[bibr23-10778012221125498] Elliott D. E. Bjelajac P. Fallot R. D. Markoff L. S. Reed B. G. (2005). Trauma-informed or trauma-denied: Principles and implementation of trauma-informed services for women. Journal of Community Psychology, 33(4), 461–477. 10.1002/jcop.20063

[bibr24-10778012221125498] Featherstone B. Morris K. White S. (2014). A marriage made in hell: Early intervention meets child protection. British Journal of Social Work, 44(7), 1735–1749. 10.1093/bjsw/bct052

[bibr10-10778012221125498] Frances T. (2022). Feminist listening and becoming: voice poems as a method of working with young women’s stories of domestic abuse in childhood. Qualitative Research in Psychology. 10.1080/14780887.2022.2071785

[bibr11-10778012221125498] Frances T. Gabriel L. James H. (2019). Young children’s narrations of relational recovery: A school-based group for children who have experienced domestic violence. Journal of Family Violence, 34(6), 565–575. 10.1007/s10896-018-0028-7

[bibr12-10778012221125498] Frances T. Turley E. Lazard L. Thompson L. Donnelly L. C. (2021). An intersectional feminist response to the UK government’s violence against women and girls 2021–2024 strategy consultation. Psychology of Women and Equalities Review, 4(2), 6–16.

[bibr25-10778012221125498] Frank A. W. (2010). Letting stories breathe: A socio-narratology. University of Chicago Press.

[bibr26-10778012221125498] Fricker M. (2007). Epistemic injustice: Power and the ethics of knowing. Oxford University Press.

[bibr27-10778012221125498] Fusco R. A. (2017). Socioemotional problems in children exposed to intimate partner violence: Mediating effects of attachment and family supports. Journal of Interpersonal Violence, 32(16), 2515–2532. 10.1177/0886260515593545 26209306

[bibr28-10778012221125498] Gill R. Scharff C. (2011). New femininities: Postfeminism, neoliberalism, and subjectivity. Palgrave Macmillan.

[bibr29-10778012221125498] Gilligan C. (2015). The listening guide method of psychological inquiry. Qualitative Psychology, 2(1), 69–77. 10.1037/qup0000023

[bibr30-10778012221125498] Gonzales G. Chronister K. M. Linville D. Knoble N. B. (2012). Experiencing parental violence: A qualitative examination of adult men’s resilience. Psychology of Violence, 2(1), 90–103. 10.1037/a0026372

[bibr31-10778012221125498] Hawkins S. Taylor K. (2015). *The changing landscape of domestic and sexual violence services: All-parliamentary group on domestic and sexual violence inquiry*. Women's Aid Federation of England. Retrieved from September 5, https://rapecrisis.org.uk/pdfs/2308_appg-changing-landscape-report-2015pdf

[bibr32-10778012221125498] Hermans H. J. M. (2001). The dialogical self: Toward a theory of personal and cultural positioning. Culture & Psychology, 7(3), 243–281. 10.1177/1354067X0173001

[bibr33-10778012221125498] HM Government. (2021). *Tackling violence against women and girls strategy*. Home Office. Retrieved from September 5, https://assets.publishing.service.gov.uk/government/uploads/system/uploads/attachment_data/file/1005074/Tackling_Violence_Against_Women_and_Girls_Strategy_-_July_2021.pdf

[bibr34-10778012221125498] Holmes M. (2004). Feeling beyond rules: Politicizing the sociology of emotion and anger in feminist politics. European Journal of Social Theory, 7(2), 209–227. 10.1177/1368431004041752

[bibr35-10778012221125498] Holt S. Buckley H. Whelan S. (2008). The impact of exposure to domestic violence on children and young people: A review of the literature. Child Abuse & Neglect, 32(8), 797–810. 10.1016/J.CHIABU.2008.02.004 18752848

[bibr36-10778012221125498] Howarth E. Moore T. H. M. Shaw A. R. G. Welton N. J. Feder G. S. Hester M. Stanley N. (2015). The effectiveness of targeted interventions for children exposed to domestic violence: Measuring success in ways that matter to children, parents and professionals. Child Abuse Review, 24(4), 297–310. 10.1002/car.2408

[bibr37-10778012221125498] Howell K. H. (2011). Resilience and psychopathology in children exposed to family violence. Aggression and Violent Behavior, 16(6), 562–569. 10.1016/J.AVB.2011.09.001

[bibr38-10778012221125498] Howell K. H. Miller-Graff L. E. (2014). Protective factors associated with resilient functioning in young adulthood after childhood exposure to violence. Child Abuse & Neglect, 38(12), 1985–1994. 10.1016/J.CHIABU.2014.10.010 25459988

[bibr39-10778012221125498] Hydén M. (2014). The teller-focused interview: Interviewing as a relational practice. Qualitative Social Work: Research and Practice, 13(6), 795–812. 10.1177/1473325013506247

[bibr40-10778012221125498] Jenney A. Alaggia R. Niepage M. (2016). “The lie is that it's not going to get better”: Narratives of resilience from childhood exposure to intimate partner violence. International Journal of Child and Adolescent Resilience, 4(1), 64–76.

[bibr41-10778012221125498] Katz E. (2015). Recovery-Promoters: Ways in which children and mothers support one another’s recoveries from domestic violence. British Journal of Social Work, 45(suppl 1), i153–i169. 10.1093/bjsw/bcv091

[bibr42-10778012221125498] Lee J. Kolomer S. Thomsen D. (2012). Evaluating the effectiveness of an intervention for children exposed to domestic violence: A preliminary program evaluation. Child and Adolescent Social Work Journal, 29(5), 357–372. 10.1007/s10560-012-0265-1

[bibr43-10778012221125498] Lenz Taguchi H. (2012). A diffractive and Deleuzian approach to analysing interview data. Feminist Theory, 13(3), 265–281. 10.1177/1464700112456001

[bibr44-10778012221125498] Livholts M. Tamboukou M. (2015). Discourse and narrative methods: Theoretical departures, analytical strategies and situated writings. Sage.

[bibr45-10778012221125498] MacQueen S. Norris P. A. (2016). Police awareness and involvement in cases of domestic and partner abuse. Policing and Society, 26(1), 55–76. 10.1080/10439463.2014.922084

[bibr46-10778012221125498] Macvarish J. Lee E. Lowe P. (2015). Neuroscience and family policy: What becomes of the parent? Critical Social Policy, 35(2), 248–269. 10.1177/0261018315574019

[bibr47-10778012221125498] McRobbie A. (2004). Post-feminism and popular culture. Feminist Media Studies, 4(3), 255–264. 10.1080/1468077042000309937

[bibr48-10778012221125498] Mullender A. Hague G. Imam U. Kelly L. Malos E. Regan L. (2002). Children’s perspectives on domestic violence. Sage.

[bibr49-10778012221125498] O’Brien K. L. Cohen L. Pooley J. A. Taylor M. F. (2013). Lifting the domestic violence cloak of silence: Resilient Australian women’s reflected memories of their childhood experiences of witnessing domestic violence. Journal of Family Violence, 28(1), 95–108. 10.1007/s10896-012-9484-7

[bibr50-10778012221125498] Orgad S. (2009). The survivor in contemporary culture and public discourse: A genealogy. The Communication Review, 12(2), 132–161. 10.1080/10714420902921168

[bibr51-10778012221125498] Ovenden G. (2012). Young women’s management of victim and survivor identities. Culture, Health & Sexuality, 14(8), 941–954. 10.1080/13691058.2012.710762 22943483

[bibr52-10778012221125498] Överlien C. Hydén M. (2009). Children’s actions when experiencing domestic violence. Childhood (Copenhagen, Denmark), 16(4), 479–496. 10.1177/0907568209343757

[bibr53-10778012221125498] Pernebo K. Almqvist K. (2016). Young children’s experiences of participating in group treatment for children exposed to intimate partner violence: A qualitative study. Clinical Child Psychology and Psychiatry, 21(1), 119–132. 10.1177/1359104514558432 25410886

[bibr54-10778012221125498] Radford L. Corral S. Bradley C. Fisher H. L. (2013). The prevalence and impact of child maltreatment and other types of victimization in the UK: Findings from a population survey of caregivers, children and young people and young adults. Child Abuse & Neglect, 37(10), 801–813. 10.1016/j.chiabu.2013.02.004 23522961

[bibr55-10778012221125498] Reich N. M. (2002). Towards a rearticulation of women-as-victims: A thematic analysis of the construction of women’s identities surrounding gendered violence. Communication Quarterly, 50(3–4), 292–311. 10.1080/01463370209385665

[bibr56-10778012221125498] Riessman C. K. (2008). Narrative methods for the human sciences. Sage Publications.

[bibr57-10778012221125498] Rose N. (1985). The psychological complex: psychology, politics, and society in England, 1869–1939. Routledge & Kegan Paul.

[bibr58-10778012221125498] Rose N. (2010). ‘Screen and intervene’: Governing risky brains. History of the Human Sciences, 23(1), 79–105. 10.1177/0952695109352415 20518155

[bibr59-10778012221125498] Rothschild B. (2017). The body remembers: Revolutionizing trauma treatment (Vol. 2). W. W. Norton & Company.

[bibr60-10778012221125498] Sanders-McDonagh E. Neville L. Nolas S.-M. (2016). From pillar to post: Understanding the victimisation of women and children who experience domestic violence in an age of austerity. Feminist Review, 112(1), 60–76. 10.1057/fr.2015.51

[bibr61-10778012221125498] Sermijn J. Devlieger P. Loots G. (2008). The narrative construction of the self: Selfhood as a rhizomatic story. Qualitative Inquiry, 14(4), 632–650. 10.1177/1077800408314356

[bibr62-10778012221125498] Smith E. Belton E. Barnard M. Fisher H. L. Taylor J. (2015). Strengthening the mother-child relationship following domestic abuse: Service evaluation. Child Abuse Review, 24(4), 261–273. 10.1002/car.2405

[bibr63-10778012221125498] Thompson L. Rickett B. Day K. (2018). Feminist relational discourse analysis: Putting the personal in the political in feminist research. Qualitative Research in Psychology, 15(1), 93–115. 10.1080/14780887.2017.1393586

[bibr64-10778012221125498] Uttal W. (2011). Mind and brain: A critical appraisal of cognitive neuroscience. The MIT Press.

[bibr65-10778012221125498] Walby S. Towers J. (2017). Measuring violence to end violence: Mainstreaming gender. Journal of Gender-Based Violence, 1(1), 11–31. 10.1332/239868017X14913081639155

[bibr66-10778012221125498] Walkley M. Cox T. L. (2013). Building trauma-informed schools and communities. Children & Schools, 35(2), 123–126. 10.1093/cs/cdt007

[bibr67-10778012221125498] Wastell D. White S. (2012). Blinded by neuroscience: Social policy, the family and the infant brain. Families, Relationships and Societies, 1(3), 397–414. 10.1332/204674312X656301

[bibr68-10778012221125498] Woodiwiss J. (2007). Politics, responsibility and adult victims of childhood sexual abuse. Sociological Research Online, 12(2), 1–12. 10.5153/sro.1404

[bibr69-10778012221125498] Woodiwiss J. (2014). Beyond a single story: The importance of separating ‘harm’ from ‘wrongfulness’ and ‘sexual innocence’ from ‘childhood’ in contemporary narratives of childhood sexual abuse. Sexualities, 17(1–2), 139–158. 10.1177/1363460713511104

[bibr70-10778012221125498] Woodiwiss J. Smith K. Lockwood K. (2017). Feminist narrative research: Opportunities and challenges. Palgrave Macmillan UK.

[bibr71-10778012221125498] Zaleski K. L. Johnson D. K. Klein J. T. (2016). Grounding Judith Herman’s trauma theory within interpersonal neuroscience and evidence-based practice modalities for trauma treatment. Smith College Studies in Social Work, 86(4), 377–393. 10.1080/00377317.2016.1222110

